# The association between S100A13 and HMGA1 in the modulation of thyroid cancer proliferation and invasion

**DOI:** 10.1186/s12967-016-0824-x

**Published:** 2016-03-23

**Authors:** Jing Zhong, Chang Liu, Ya-jun Chen, Qing-hai Zhang, Jing Yang, Xuan Kang, Si-Rui Chen, Ge-bo Wen, Xu-yu Zu, Ren-xian Cao

**Affiliations:** Institute of Clinical Medicine, The First Affiliated Hospital of University of South China, 421001 Hengyang, Hunan People’s Republic of China; Department of Metabolism and Endocrinology, The First People’s Hospital of Chenzhou, Luojiajing Road, 102, 423000 Chenzhou, Hunan People’s Republic of China; Department of Metabolism and Endocrinology, The Second Affiliated Hospital of University of South China, 421001 Hengyang, Hunan People’s Republic of China; Department of Metabolism and Endocrinology, The First Affiliated Hospital of University of South China, 421001 Hengyang, Hunan People’s Republic of China

**Keywords:** S100A13, HMGA1, RNA interference, Proliferation, Invasion, Thyroid cancer

## Abstract

**Background:**

S100A13 and high mobility group A (HMGA1) are known to play essential roles in the carcinogenesis and progression of cancer. However, the correlation between S100A13 and HMGA1 during cancer progression is not yet well understood. In this study, we determined the effects of S100A13 on HMGA1 expression in thyroid cancer cells and examined the role of HMGA1 in thyroid cancer progression.

**Methods:**

Stable ectopic S100A13 expression TT cellular proliferation was evaluated by nude mice xenografts assays. The effect of lentivirus-mediated S100A13 knockdown on thyroid cancer cellular oncogenic properties were evaluated by MTT, colony formation assays and transwell assays in TPC1 and SW579 cells. The effect of siRNA-mediated HMGA1 knockdown on thyroid cancer cellular proliferation and invasion were evaluated by MTT, colony formation assays and transwell assays. The tissue microarray was performed to investigate the correlation between S100A13 and HMGA1 expression in tumor tissues.

**Results:**

The ectopic expression of S100A13 could *increase tumor growth in a TT cell xenograft mouse model.* Moreover, lentivirus-mediated S100A13 knockdown led to the inhibition of cellular oncogenic properties in thyroid cancer cells, and HMGA1 was found to be involved in the effect of S100A13 on thyroid cancer growth and invasion. Furthermore, siRNA-mediated HMGA1 knockdown was proved to inhibit the growth of TPC1 cells and invasive abilities of SW579 cells. Clinically, it was revealed that both S100A13 and HMGA1 showed a higher expression levels in thyroid cancer cases compared with those in matched normal thyroid cases *(P* = *0.007 and P* = *0.000)*; S100A13 and HMGA1 expressions were identified to be positively correlated *(P* = *0.004, R* = *0.316)* when analyzed regardless of thyroid cancer types.

**Conclusions:**

This is the first report for the association between HMGA1 and S100A13 expression in the modulation of thyroid cancer growth and invasion. Those results would provide an essential insight into the effect of S100A13 on carcinogenesis of thyroid tumor, rending S100A13 to be potential biological marker for the diagnosis of thyroid cancer.

**Electronic supplementary material:**

The online version of this article (doi:10.1186/s12967-016-0824-x) contains supplementary material, which is available to authorized users.

## Background

S100A13 is a small calcium (Ca^2+^)-binding protein which belongs to the S100 family. It is characterized by its specificity for diverse forms of cancer [1–3]. S100A13 could regulate secretion of FGF1 and IL1α and could be a marker for vessel density and on a cellular level [4, 5]. In addition S100A13 is also involved in cell cycle progression and differentiation, including cytokine and NF-κB signalling, suggesting that S100A13 may be related to increased aggressiveness of melanoma tumours [6, 7]. Several studies reported positive correlation between the elevated expression of S100A13 and risk of relapse and status of melanoma patients at follow-up, indicating that S100A13 may play a crucial role in melanoma chemoresistance [8, 9]. Moreover, S100A13 was found to be involved in the invasiveness of lung cancer cell lines [10], and also was detected in tumor cells circulating in blood of patients with metastatic cancer [11], indicating that this protein may be considered as a predictor of cancer metastases. Recently, a study on cystic papillary thyroid carcimoma (cPTC) confirmed a significant up-regulation of cytokeratin 19 and S100A13 in cPTC compared to benign lesions, suggesting their possible use in fine needle aspiration biopsy based preoperative diagnostics of cystic thyroid lesions [12]. With multiplexed and targeted mass spectrometry method, S100A13 was also found to be elevated in papillary thyroid carcinoma (PTC) compared to normal tissue, suggesting that S100A13 may be considered as a novel candidate PTC biomarker [13].

High mobility group A (HMGA1) is an architectural transcription factor that encodes a nonhistone chromatin protein. Two isoforms, HMGA1a and HMGA1b are produced which do not show direct transcriptional regulation activities, but they regulate the transcriptional activity of several genes by altering the chromatin structure [14–16]. The expression of HMGA1 is absent or present only at low level in normal cell and adult tissues but are elevated in embryonic cells [17] and many malignant neoplasias, including breast [18], pancreas [19], lung [20], ovary [21], colon [22] and thyroid carcinomas [23]. HMGA1 has been confirmed to associate with the initiation and progression of diverse types of tumors [24–27]. HMGA1 has also been reported to correlate with the presence of metastasis and reduced survival [28], and could be a poor prognostic marker.

In this study, we examined the effects of S100A13 and HMGA1 on thyroid cancer progression. The results suggest that HMGA1 was involved in the effect of S100A13 on thyroid cancer growth and invasion by modulating the expression of Snail and E-cadherin. In addition, a tissue microarray revealed that higher expressions of both S100A13 and HMGA1 were observed in thyroid cancer cases compared with that in normal thyroid cases. Statistical analysis also confirmed that S100A13 and HMGA1 expressions were positively correlated. This study establish the first link between S100A13 and HMGA1 in thyroid cancer, providing further evidence of the pivotal role of HMGA1 in thyroid cancer progression.

## Methods

### Cell culture

Human thyroid cancer cell line TT, TPC1 and SW579 were purchased from American Type Culture Collection (USA). After thawing, TT cells were cultured in F12 K (N3520; Sigma, USA) medium supplemented with 10 % fetal bovine serum (Gibco, USA) and NaHCO_3_ 2.5 g/L at 37 °C in a humidified atmosphere containing 5 % CO_2_. TPC1 cells were cultured in DMEM medium supplemented with 10 % fetal bovine serum at 37 °C in a humidified atmosphere containing 5 % CO_2_. SW579 cells were cultured in L15 medium supplemented with 10 % fetal bovine serum at 37 °C in a humidified atmosphere without CO_2_. After three passages, cells were used for viral infection.

### Western blot analysis

Total cell lysates were lysed on ice for 30 min. Soluble proteins (20 μg) were probed with anti-S100A13, anti-HMGA1, anti-Snail, and anti-E-cadherin antibodies (1:500, Abcam). Loading variations were normalized against β-actin, which was identified by anti-β-actin monoclonal antibody (1:1000, Abcam).

### Construction and screening of lentiviral vectors harboring S100A13-specific siRNA

The siRNA sequences targeting to human S100A13 gene (GenBank accession No. NM_001024210) were selected: Target1: ATGAGTACTGGAGATTGAT; Target2: CTCGGAGCTCAAGTTCAAT; and Target3: TGGGCTCTCTTGATGAGAA. Three pairs of complementary oligonucleotides were then designed (Additional file 1: Table S1). The stem-loop oligonucleotides were synthesized and cloned into a lentivirus-based vector carrying the green fluorescent protein (GFP) gene (pGCSIL-GFP, Genechem, Shanghai, China). A universal sequence (PSC-NC: TTCTCCGAACGTGTCACGT) was used as a negative control for RNA interference. Lentiviral particles were prepared as previously described [29].

Three siRNA-carrying lentiviral vector constructs were used to infect TPC1 at a multiplicity of infection (MOI) of 20 (low MOI) and 40 (high MOI). Three days after infection, GFP expression was detected to calculate the infection efficiency. Five days after infection, cells were harvested. Real-time reverse transcription polymerase chain reaction (RT-PCR) was performed to determine S100A13 knockdown efficiency and screen for the siRNA with the highest knockdown efficiency which was then used for subsequent experiments.

### RNA isolation and reverse transcription PCR (RT-PCR)

The SW579 were treated with Scramble RNA or HMGA1 siRNA (40, 80, 160 nM), and maintained in culture medium for 48 h. Total RNA was extracted from the SW579 cells using TRIzol reagent (Invitrogen) and the total RNA was reverse transcribed into cDNA using the first-strand synthesis kit (Gibco-BRL, Carlsbad, CA, USA). The mRNAs of HMGA1, E-cadherin, Snail and β-actin were amplified using the primers (Additional file 2: Table S2). The gene-specific primers were amplified with a denaturation step (95 °C for 2 min), followed by 35 cycles of denaturation (95 °C for 30 s), annealing (55 °C for 30 s) and extension (72 °C for 50 s). Samples from three separate experiments were analyzed in duplicate. The results from RT-PCR were expressed using β-actin as a reference.

### Cell proliferation and colony formation assays

For cell proliferation assays, cells were seeded in a 96-well plate (2000 cells/well) and counted using an automated cell counter (Nexcelom Bioscience, Lawrence, MA, USA). For colony formation assay, cells were seeded in a 12-well plate (400 cells/well) and maintained for 8 days. Each experiment was carried out in triplicate and performed at least twice.

### Cell invasion assays

For the invasion assays, 10,000 cells were resuspended in serum-free medium and placed in the upper chamber of a 24-well Matrigel™ Invasion Chamber (BD Biosciences, SanDiego, CA, USA) coated with Matrigel. Cell invasion was calculated as the percentage of total cells that had invaded the bottom chamber containing complete medium with serum.

### Scratch-wound assays

For the scratch-wound assays, cells were transiently transfected with shRNA vectors and grown to confluence. A central linear wound area was carefully created by scraping the cell monolayer with a sterile 200 μl pipette tip, and images were taken after 24 h. Bars represent as the mean percentage of wound closure relative to the initial wound area.

### Tumour xenografts

Four-week-old BALB/c female nude mice were fed on the super-clean biological laminar flow shelf for 1 week. All of the in vivo experimental protocols were approved by the Animal Care Committee of South China University. The details of tumour xenografts assay were performed as described previously [30].

### Transient transfection and luciferase activity assay

Transient gene delivery was carried out to assess the effect of HMGA1 on Snail and E-cadherin promoter activity in SW579 cells as described previously [31]. A luciferase assay kit (Promega) was used to measure the reporter activity according to the manufacturer’s instructions. Luciferase activity was normalized by using a Renilla luciferase internal control.

### Tissue microarray and immunohistochemical analysis

The tissue microarray (TH8010, US Biomax) consisting 70 thyroid cancer cases and 10 normal cases was utilized, and was histologically interpretable and analyzed for the correlation with clinicopathological parameters. IHC staining was performed as detailed in our previous studies [30]. The mouse monoclonal S100A13 (1:25; ab55701, Abcam) antibody and the rabbit polyclonal HMGA1 antibody (1:150; ab4078, Abcam) were used.

### Statistical analysis

All experiments were performed with three replicates and the results were expressed as the mean ± S.E.M or mean ± SD. Statistical analysis was done using SPSS, version 13.0. A statistical association between clinicopathological and molecular parameters was tested, using non-parametrically two-tailed Mann–Whitney U test. P values < 0.05 were considered significant. Spearman’s rank-correlation coefficients were used to assess the relationship between *S100A13* and *HMGA1* expression.

## Results

### S100A13 overexpression increases tumor growth in a TT cell xenograft mouse model

**Fig. 1:**
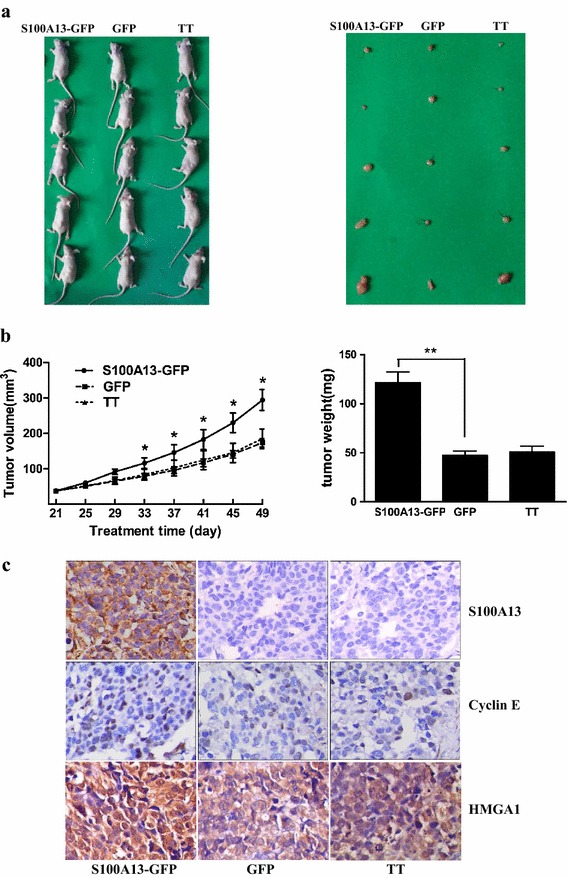
S100A13 overexpression increases tumor growth in a TT cell xenograft mouse model. **a** S100A13-GFP, GFP or TT groups cells were transplanted into ovariectomized athymic mice. *Left* mice appearances in different treated groups. *Right* tumor appearances in different treated groups. **b** *Left* increased tumor volume in S100A13 overexpression mice. Tumours were measured weekly using a vernier calliper and the volume was calculated according to the formula: π/6 × length × width^2^. Each *point* represents the mean ± SD for different animal measurements (n = 5) (*P < *0.05*). *Right* increased tumor weight in S100A13 overexpression mice (**P < *0.01*). **c** Detection of proteins expression by immunohistochemical assay of tumor tissues in nude mice in different treated groups (streptavidin biotin complex ×400)

It was previously revealed that S100A13 had essential roles in various cancer, we therefore exploited whether S100A13 was involved in the development of thyroid cancer. Using Stable cell lines with the GFP and S100A13-GFP, we found that overexpression of S100A13 in the transfected thyroid cancer TT cells markedly increased cells proliferate ability, and decreased population percentage of G_0_/G_1_ period compared to those cells with either GFP or untransfected TT cells [32]. To further examine the effects of S100A13 on cell proliferation in vivo, we transplanted three types of thyroid tumours cells developed from TT cells (S100A13-GFP, GFP and TT) into nude mice. Growth of the implanted tumours was measured in mice (n = 5 for each group) over a period of 7 weeks. Overexpression of S100A13 dramatically increased the size and weight of tumors compared to those engrafted with GFP cells or with untransfected TT cells (*P* < *0.05 and P* < *0.01*, Fig. 1a, b), but the difference between the GFP cells group and untransfected TT cells group was not significant. Cyclin E, one of the key gene of cell cycle was found to be upregulated in S100A13-GFP cells (Fig. 1c), indicating that it might be involved in S100A13 induced cell proliferation. HMGA1, an architectural transcription factor associating with the initiation and progression of diverse types of tumors, also was found to be upregulated in S100A13-GFP cells (Fig. 1c). Those data suggest that overexpression of S100A13 enhances oncogenic properties of thyroid cancer cells in vitro and in vivo.

### S100A13 knockdown inhibits in vitro cell growth in the least/non-invasive cell line

**Fig. 2:**
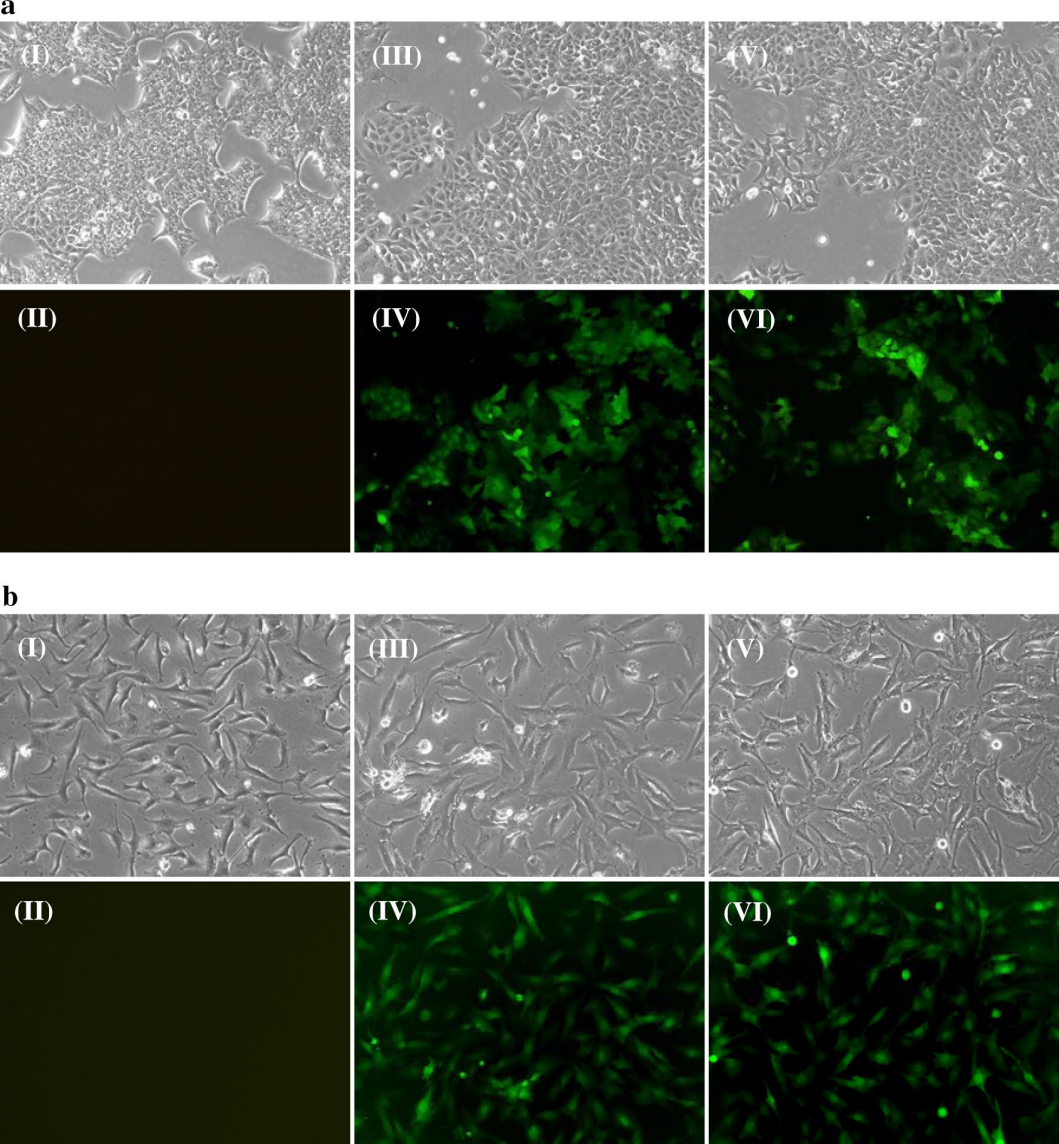
**a** Fluorescence microscopy examination of the infection efficiencies of different lentiviral vectors in TPC1 cells (magnification ×100). I, TPC1 cells without lentiviral infection (Con group) in the light microscope; II, TPC1 cells of Con group in the fluorescence microscope; III, TPC1 cells were infected with negative lentivirus NC/GV248RNAi-LV (NC group) in the light microscope; IV, TPC1 cells of NC group in the fluorescence microscope; V, TPC1 cells were infected with lentivirus S100A13/GV248RNAi-LV#1 RNAi (KD group) at a high MOI in the light microscope; VI, TPC1 cells of KD group at a high MOI in the fluorescence microscope. **b** Fluorescence microscopy examination of the infection efficiencies of different lentiviral vectors in SW579 cells (magnification ×100). The description of* panels* was similar with that in** a**

**Fig. 3:**
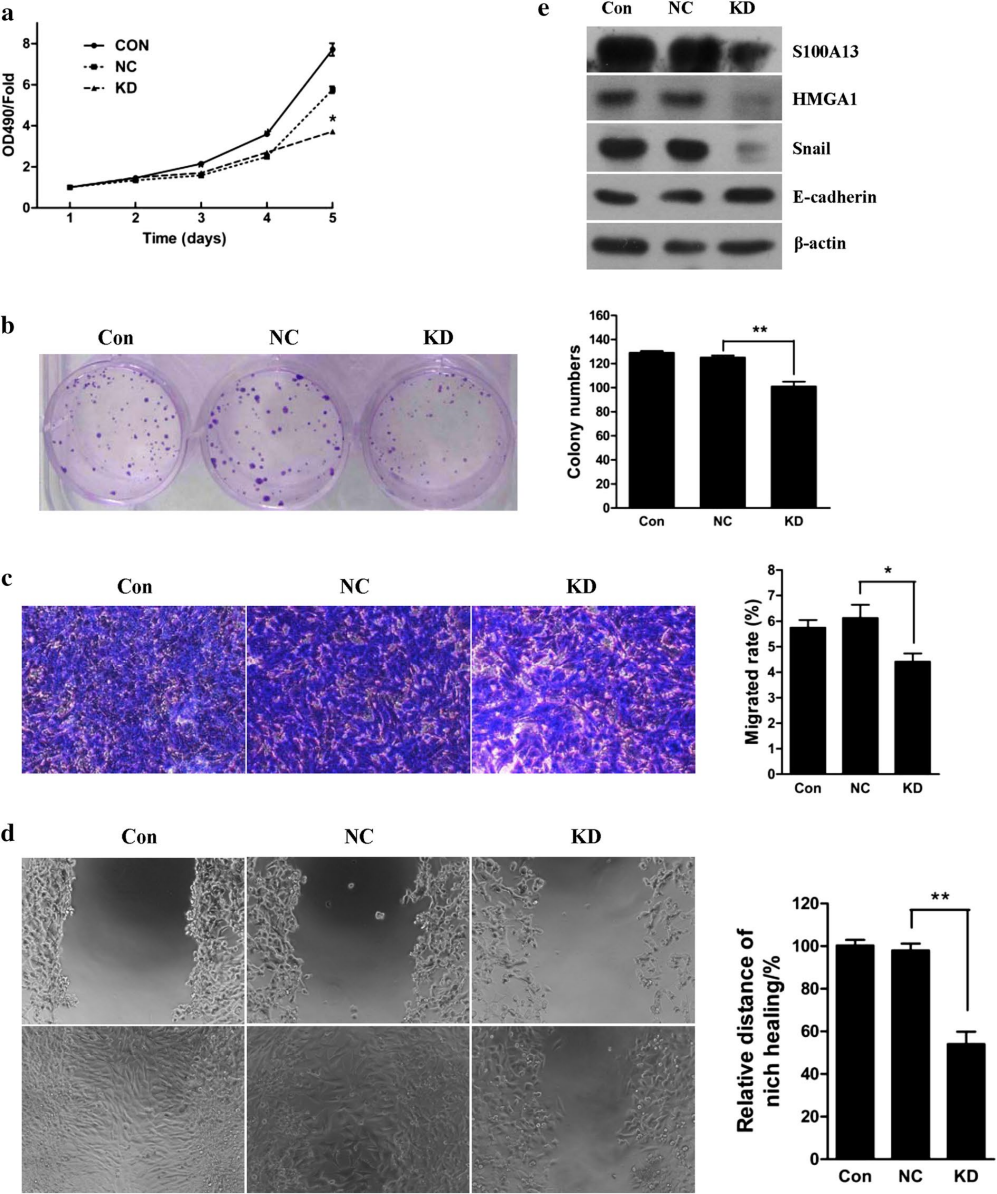
Lentivirus-mediated S100A13 knockdown inhibited thyroid cancer cell proliferation and invasion. **a** Growth curves of cells in each group. MTT analysis showed that lentivirus-mediated S100A13 knockdown significantly inhibited thyroid cancer TPC1 cell proliferation (n = 3). *P < *0.05.* **b** *Colony formation* assay showed that lentivirus-mediated S100A13 knockdown significantly inhibited thyroid cancer TPC1 cell colony formation (n = 3, **P < *0.01).* **c** *Transwell invasion* assay showed that lentivirus-mediated S100A13 knockdown significantly inhibited thyroid cancer SW579 cell invasion. Cells were transiently transfected with lentivirus-mediated shRNA plasmids and plated on the top of the transwells. Twenty-four hours after plating, cells invaded through the pores were counted. Values are expressed as mean ± SD of three independent experiments, *P < *0.05.* **d** *Scratch-wound* assay showed that lentivirus-mediated S100A13 knockdown significantly inhibited thyroid cancer SW579 cell migration. **e** *Western blot* assay showed that lentivirus-mediated S100A13 knockdown inhibited expression of HMGA1 and Snail, promoted expression of E-cadherin in SW579 cells

S100A13/GV248RNAi-LV-1 was transfected into thyroid cancer TPC1 cells and SW579 cells, individually. The infection efficiencies of these lentiviral vectors were all above 90 % as revealed by fluorescence microscopy (Fig. 2a, b). Real-time RT-PCR assay showed that all three constructs, whether they were used at a high or low MOI, could significantly downregulate S100A13 gene expression in TPC1 cells (Additional file 3: Figure S1). As shown in Fig. 3a, the MTT assay showed that TPC1 cell proliferation was significantly inhibited in the S100A13 knockdown group as compared to those of the control group and the NC group at the fifth days (*P* < *0.05)*, however, the SW579 cell proliferate ability of S100A13 knockdown group showed no significant difference compared to those of the control group and the NC group at the indicated time points (Additional file 4: Figure S2a).

Colony formation assay showed that the number of formed colonies from TPC1 cells with lentivirus-mediated S100A13 knockdown was markedly decreased compared to those of the control group and the NC group (Fig. 3b, *P* < *0.01*). Whereas, the number of formed colonies of SW579 cells carrying with lentivirus-mediated S100A13 knockdown showed no significant difference to those of the control group and NC group (Additional file 4: Figure S2b). Those results indicated that S100A13 knockdown could inhibit the cell proliferation and colony formation of the least/non-invasive thyroid cancer TPC1 cells, but show no obvious effect on the proliferate ability of invasive thyroid cancer SW579 cells.

### S100A13 knockdown inhibits the invasive and migration capabilities through decrease the expression of HMGA1 in thyroid cancer SW579 cells

To determine if S100A13 knockdown was able to affect the invasion properties of thyroid cancer TPC1 cells and SW579 cells, the transwell invasion assay and scratch-wound assay were performed. As shown in Fig. 3c, compared to those of the control group and the NC group, the invasion rate of SW579 cells in the S100A13 knockdown group decreased significantly (*P* < *0.05)*. As well as the result of invasion assay, the migration capability of the S100A13 knockdown group decreased significantly compared to those of the control group and the NC group (Fig. 3d, *P* < *0.01*). The invasive and migration capabilities showed no significant difference between the control group and the NC group (Fig. 3c, d). The thyroid cancer TPC1 cells, however, showed no invasive capability even in the control group, and the migration capability of TPC1 cell with the S100A13 knockdown group showed no obvious difference compared to those of the NC group (Additional file 5: Figure S3). To determine if S100A13 knockdown was able to affect the expressions of invasion associated factors, the western blot was performed. As shown in Fig. 3e, S100A13 knockdown could lead to the downregulation of HMGA1 and Snail and the upregulation of E-cadherin in SW579 cells. Moreover, the ectopic S100A13 expression could increase both HMGA1 and Snail mRNA expressions (Additional file 6: Figure S4) and enhance the promoter activities (Additional file 7: Figure S5).

### HMGA1 knockdown inhibits in vitro cell growth and invasion

**Fig. 4:**
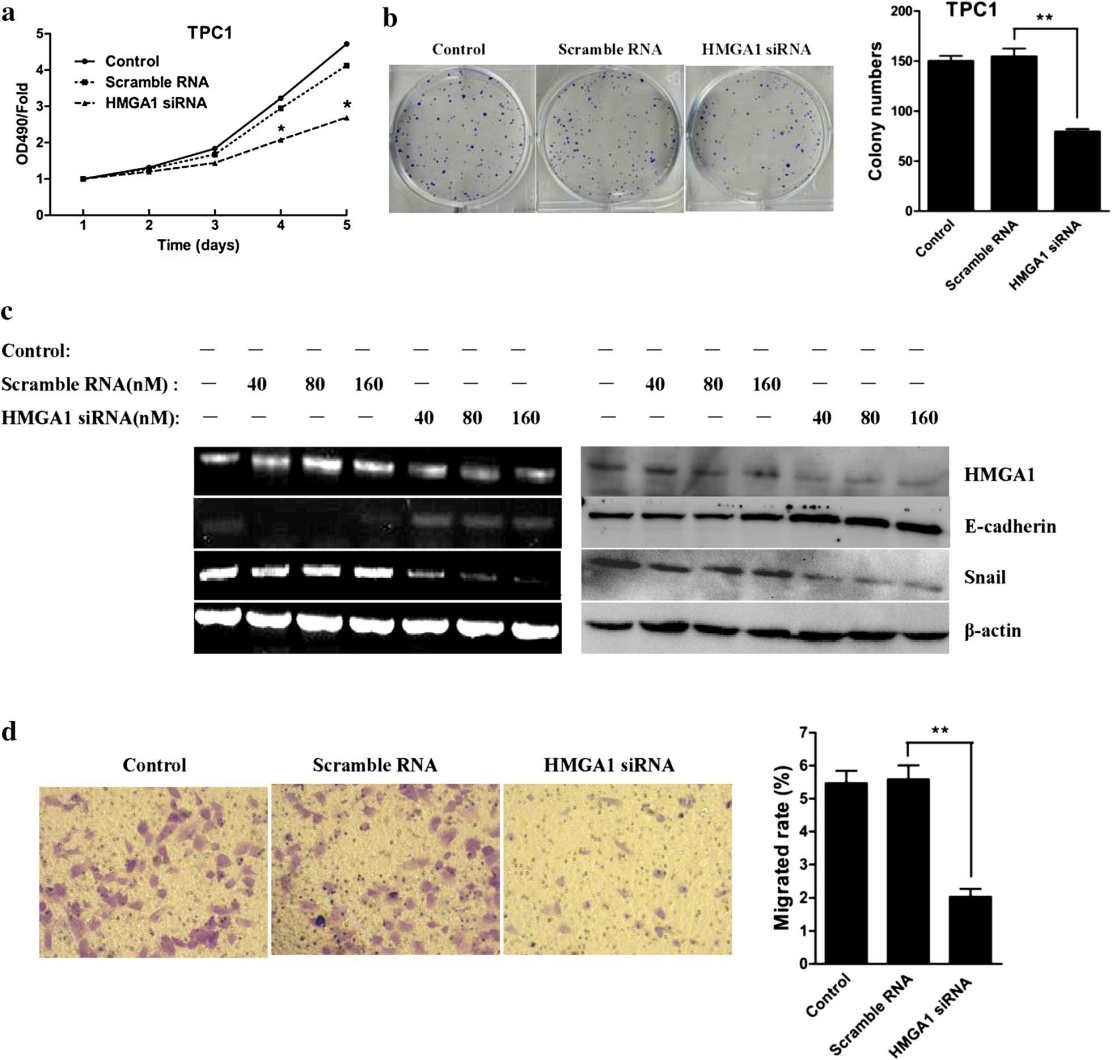
SiRNA-mediated HMGA1 knockdown inhibited thyroid cancer cell proliferation and invasion. **a** Growth curves of cells in each group. MTT analysis showed that siRNA-mediated HMGA1 knockdown significantly inhibited thyroid cancer TPC1 cell proliferation (n = 3). *P < *0.05.* **b** *Colony formation* assay showed that siRNA-mediated HMGA1 knockdown significantly inhibited thyroid cancer TPC1 cell colony formation (n = 3, **P < *0.01).* **c** RT-PCR and *western blot* assay showed that siRNA-mediated HMGA1 knockdown inhibited mRNA and protein expression of HMGA1 and Snail, promoted mRNA and protein expression of E-cadherin in thyroid cancer SW579 cell. **d** *Transwell invasion* assay showed that siRNA-mediated HMGA1 knockdown significantly inhibited thyroid cancer SW579 cell invasion. Cells were transiently transfected with HMGA1-siRNA and plated on the top of the transwells. Twenty-four hours after plating, cells invaded through the pores were counted. Values are expressed as mean ± SD of three independent experiments, **P < *0.01*

It was reported that HMGA1 overexpression could promote the growth and invasion of cancer cells [24–27]. To address the effect of HMGA1 on thyroid cancer cells, the HMGA1 targeting siRNA were used. As shown in Fig. 4a and b, HMGA1 silence in TPC-1 cell could inhibit the cell proliferation and decrease the colony formation ability. Furthermore, it was revealed that the siRNA induced HMGA1 could lead to the downregulation of Snail and upregulation of E-cadherin in both mRNA or protein levels in SW579 cells (Fig. 4c), resulting in the decrease of invasive abilities of thyroid cancer SW579 cells (Fig. 4d, *P* < *0.01*).

**Fig. 5:**
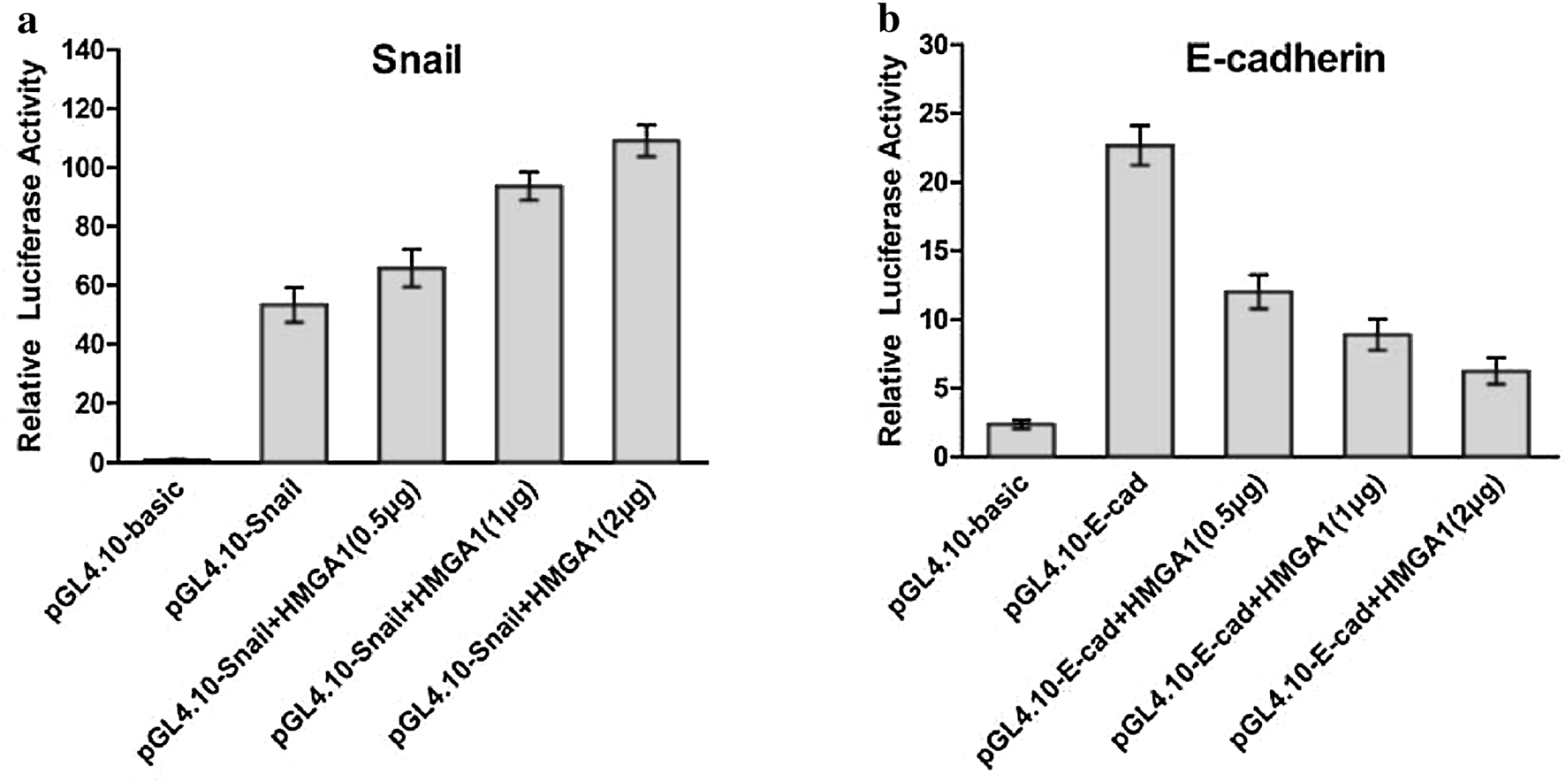
HMGA1 overexpression affect Snail and E-candherin promoter activities in SW579 cell. **a, b** Luciferase activity assay showed that HMGA1 overexpression inhibited promoter activity of E-cadherin, promoted promoter activity of Snail in a dose-dependent manner

To further clarify the mechanism by which HMGA1 affect the expression of Snail or E-cadherin in SW579 cells, luciferase reporter assay was performed. It was demonstrated that HMGA1 could enhance the promoter activities of *Snail* gene and inhibit the promoter activities of *E*-*cadherin* in a dose-dependent manner (Fig. 5a, b). Those results indicate that HMGA1 might affect the expression of E-cadherin and Snail by regulating the promoters’ activities in SW579 cells.

### S100A13 correlates with HMGA1 expression in thyroid carcinoma

**Fig. 6:**
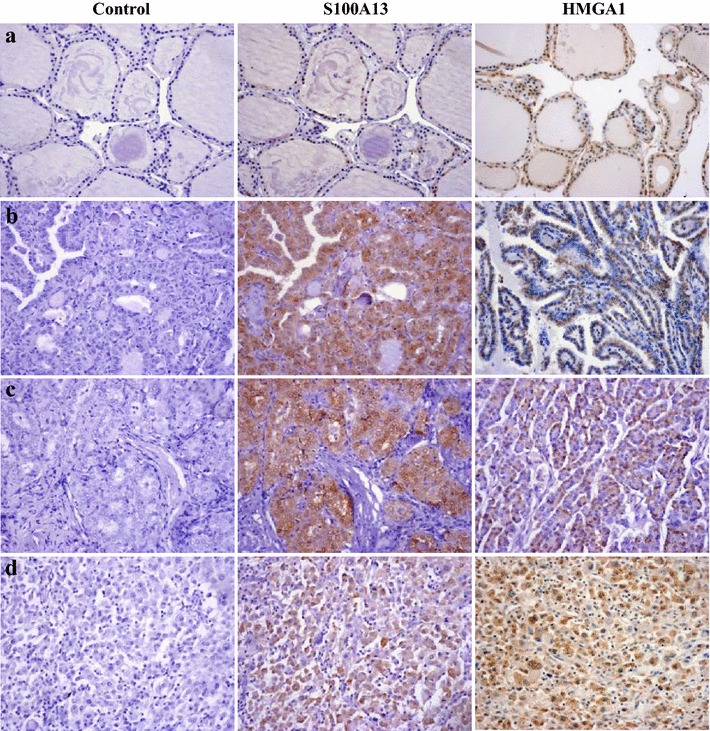
S100A13 and HMGA1 protein expression show a positive correlation in thyroid carcinoma (images in original magnification, ×200). **a** Normal thyroid tissue, **b** thyroid papillary carcinoma, **c** thyroid follicular carcinoma, **d** thyroid undifferentiated carcinoma

To further indentify the relationship between S100A13 and HMGA1 expression in thyroid cancer, tissue microarray (TH8010, US Biomax), consisting of 70 thyroid cancer cases and 10 normal cases was used, and the clinicopathologic data were available in Table 1. The immunohistochemistry analysis showed that S100A13 was stained in 94.3 % of thyroid tumors, predominantly in nuclear with faint cytoplasmic staining, and was stained in 60.0 % in normal thyroid tissue (*P* = *0.007,* Table 2). HMGA1 was stained in 98.6 % of thyroid tumors, predominantly in nuclear with faint cytoplasmic staining, and was stained in 60.0 % in normal thyroid tissue (*P* = *0.000,* Table 2). S100A13 and HMGA1 immunostainings of tumor and normal tissue of representative cases of thyroid carcinoma are shown in Fig. 6. Furthermore, S100A13 and HMGA1 expressions were found to be positively correlated (*r* = *0.316, P* = *0.004*), when analyzed regardless of thyroid cancer types (Table 3). S100A13 expressions in thyroid carcinoma did not show a statistically significant correlation with patient age and nodal metastasis of the tumor (Table 1, *P* > *0.05*). However, S100A13 expression in thyroid carcinoma was shown to be more common in male than that in female, and much higher in papillary cancer compared with follicular cancer and undifferentiated cancer (Table 1, *P* = *0.049 and 0.051*). HMGA1 expression in thyroid carcinoma also showed a statistically significant correlation with the patient sex and tumor types (Table 1, *P* = *0.016 and 0.003*). Those tissue microarray data confirmed that S100A13 and HMGA1 expression were positively correlated in thyroid carcinoma, and it may be involved in the progression of thyroid cancer.

**Table 1:** Correlation of S100A13 and HMGA1 expression with clinicopathological parameters

	n	S100A13				P value	HMGA1			P value
		−	+	++	+++		−	+	++	
Age (years)						0.549				0.984
>50	30	2	7	20	1		0	13	17	
≤50	40	2	7	30	1		1	16	23	
Sex						0.049				0.016
Male	14	0	1	12	1		0	2	12	
Female	56	4	13	38	1		1	27	28	
Tumor types						0.051				0.003
Papillary	46	2	5	38	1		1	14	31	
Follicular	18	1	7	9	1		0	14	4	
Undifferentiated	6	1	2	3	0		0	1	5	
Node metastasis						0.213				0.063
Positive	17	0	2	15	0		0	4	13	
Negative	53	4	12	35	2		1	25	27

**Table 2:** Expressions of S100A13 and HMGA1 in thyroid normal tissue and cancer

	n	S100A13		P value	HMGA1		P value
		Positive	Negative		Positive	Negative	
Tissue types				0.007			0.000
Normal	10	6	4		6	4	
Tumor	70	66	4		69	1

**Table 3:** Correlation of S100A13 and HMGA1 expression

	n	HMGA1			Spearman (r)	P value
		−	+	++		
S100A13					0.316	0.004
−	4	1	2	1		
+	14	0	6	8		
++ ~ +++	52	0	21	31		

## Discussion

The S100 protein family has been implicated in the regulation of a number of cellular processes such as cell growth and differentiation, cell cycle progression, phosphorylation regulation of proteins, protein secretion, organization of membrane structure and cytoskeleton dynamics [33]. Recently, increasing number of studies report that S100 family members are associated with a variety of human diseases, including neurodegeneration, inflammatory disorders and cancers [34]. In particular, S100A4, S100A6, S100A7 and S100A10 have been found to be overexpressed in some cancer types, and could be associated with aggressive cancer phenotype [35–39]. However, the molecular mechanisms of S100 proteins implicating in tumor progression remain to be elucidated. S100A13 is the member of S100 family, and it has been shown to be involved in the nonclassical export of signal-peptideless proteins, including fibroblast growth factors, interleukin 1α, and synaptotagmins [40, 41]. Recent data showed that S100A13 is related to inflammatory functions [5]. It was also reported that S100A13 expressed differentially during brain development, suggesting that it may play a role in nervous system function [41–43]. Increasing evidences also showed the association between S100A13 and tumourigenesis, indicating the role of S100A13 in initial and progression in diversity of cancer [6, 7, 9–11].

In this study, experiments were performed to determine the effects of S100A13 on cell proliferation, and we demonstrated that the ectopic expression of S100A13 could enhance cellular proliferation of thyroid cancer TT cells in vivo. To further define the effect of S100A13 on other thyroid cancer cells, three lentivirus-mediated S100A13 gene targeting shRNA (KD1, KD2 and KD3) were used to silence the expression of S100A13 in thyroid cancer TPC1 and SW579 cells. It was revealed that the downregulation of S100A13 could inhibit the TPC1 cells growth in vitro and lead to the decrease of invasive and migration ability of SW579 cell line in vitro. Those findings provide evidences for the involvement of S100A13 in the modulation of thyroid cancer cell proliferation and invasion. It was reported that the IL1α-S100A13 complex plays an important role in cell proliferation and angiogenesis, and would be an effective strategy to inhibit a wide range of cancers [41]. It was also reported that S100A13 increased in human astrocytic gliomas, in which it correlates with VEGF-A expression, microvessel density and tumor grading [7] and it is also associated with a more aggressive, invasive phenotype in lung cancer-derived cell lines [10]. Moreover, S100A13 was detected in tumor cells circulating in blood of patients with metastatic cancer, indicating that S100A13 could be considered as a predictor of metastases [11].

Intriguingly, the siRNA induced S100A13 downregualtion was found to cause the decreased expression of HMGA1 and Snail and increased E-cadherin expression in SW579 cells, indicating the involvements of HMGA1, Snail and E-cadherin in S100A13 induced celluar proliferation and invasion. Recent study reports silencing HMGA1 could block proliferation, migration and invasion of triple negative breast cancer MDA-MB-231 cells. Mesenchymal genes (Vimentin, Snail) are repressed, while E-cadherin is induced in the HMGA1 knock-down cells [18]. In our study, the downregulation of HMGA1 was further confirmed to be able to decrease and increase the expression of Snail and E-cadherin respectively, resulting in the suppression of TPC1 cells proliferation and SW579 cells invasion. Those results indicate that S100A13 and HMGA1 show the consisitent effects on the proliferation and invasion of thyroid cancer cells.

The higher expressions of both S100A13 and HMGA1 were observed in thyroid cancer tissues compared with that in normal thyroid tissues through the analysis of tissue microarray, indicating that the elevated expression of S100A13 and HMGA1 in thyroid cancer tissue might play a role in the initiation and progression of thyroid cancer. Further analysis to the tissue microarray data unraveled a positive correlation between S100A13 and HMGA1 expression (*P* = *0.004*) regardless of thyroid cancer types, which is consistent with the results from cells models, suggesting that S100A13 may be of importance in regulating HMGA1 expression in human thyroid cancer, and that S100A13 might be responsible for the increased ability of proliferation and invasion in thyroid cancer cells. Moreover, the expressions of S100A13 and HMGA1 in thyroid carcinoma were shown to be associated with the patient sex and tumor types, which is not be reported in other cancers previously. The further clarification of underlying molecular events would be helpful for understanding the role of S100A13 and HMGA1 in the progression of thyroid cancer.

## Conclusions

In summary, this is the first report for the association between HMGA1 and S100A13 expression in the modulation of thyroid cancer growth and invasion. Those results would provide an essential insight into the effect of S100A13 on carcinogenesis of thyroid tumor, rending S100A13 to be potential biological marker for the diagnosis of thyroid cancer.

## Additional files

10.1186/s12967-016-0824-x The structure of siRNAs in lentiviral vectors.
10.1186/s12967-016-0824-x The sequences of siRNA and primers for RT-PCR.
10.1186/s12967-016-0824-x Selection of the lentiviral vector harboring shRNA with the highest knockdown efficiency. Relative levels of S100A13 in TPC1 cells infected with different groups of lentiviral particles at a low MOI or a high MOI. Low dose group: The dose of lentiviral plasmid was 0.30 μg; High dose group: The dose of lentiviral plasmid was 0.6 μg. ***P* *<* *0.01*, vs NC group; n = 3. The highest knockdown efficiency was achieved using KD1-harboring lentiviral particles.
10.1186/s12967-016-0824-x Lentivirus-mediated S100A13 knockdown was utilized to detect the effect on cellular proliferation with MTT (**a**) and colony formation assays (**b**) in SW579 cell.
10.1186/s12967-016-0824-x Lentivirus-mediated S100A13 knockdown was utilized to detect the effect on migration capability with scratch-wound assays in TPC1 cell.
10.1186/s12967-016-0824-x S100A13 increase the mRNA levels of HMGA1 and SNAIL in TPC-1 cells. The GV219/S100A13 plasmid was introduced into TPC-1 cells for 48 h, and the mRNA levels of HMGA1 and SNAIL were assessed by Q-PCR. Average values of three independent experiments are shown, error bar indicates ±s.d. **p < 0.05.
10.1186/s12967-016-0824-x S100A13 increase the promoter activities of *HMGA1* and *SNAIL* in TPC-1 cells. The PGL.10/ HMGA1 and PGL.10/ SNAIL were co-transfected with or without GV219/ S100A13 plasmid into TPC-1 cells. Average values of three independent experiments are shown, error bar indicates ±s.d. *p < 0.05, **p < 0.01.
