# Supplementary material for: The association between S100A13 and HMGA1 in the modulation of thyroid cancer proliferation and invasion
Source: J Transl Med. 2016 Mar 23;14:80. doi: 10.1186/s12967-016-0824-x (PMC4804518; doi:10.1186/s12967-016-0824-x)
Supplement: Supplementary file 1 — 10.1186/s12967-016-0824-x The structure of siRNAs in lentiviral vectors. [file 12967_2016_824_MOESM1_ESM.docx]

Table S1 The structure of siRNAs in lentiviral vectors

| **NO.** | **5’** | **STEM** | **Loop** | **STEM** | **3’** |
| --- | --- | --- | --- | --- | --- |
| S100A13-RNAi-a | Ccgg | caATGAGTACTGGAGATTGAT | CTCGAG | ATCAATCTCCAGTACTCATTG | TTTTTg |
| S100A13-RNAi-b | aattcaaaaa | caATGAGTACTGGAGATTGAT | CTCGAG | ATCAATCTCCAGTACTCATTG |  |
| S100A13-RNAi-a | Ccgg | gaCTCGGAGCTCAAGTTCAAT | CTCGAG | ATTGAACTTGAGCTCCGAGTC | TTTTTg |
| S100A13-RNAi-b | aattcaaaaa | gaCTCGGAGCTCAAGTTCAAT | CTCGAG | ATTGAACTTGAGCTCCGAGTC |  |
| S100A13-RNAi-a | Ccgg | tgTGGGCTCTCTTGATGAGAA | CTCGAG | TTCTCATCAAGAGAGCCCACA | TTTTTg |
| S100A13-RNAi-b | aattcaaaaa | tgTGGGCTCTCTTGATGAGAA | CTCGAG | TTCTCATCAAGAGAGCCCACA |  |
