# Supplementary material for: The association between S100A13 and HMGA1 in the modulation of thyroid cancer proliferation and invasion
Source: J Transl Med. 2016 Mar 23;14:80. doi: 10.1186/s12967-016-0824-x (PMC4804518; doi:10.1186/s12967-016-0824-x)
Supplement: Supplementary file 2 — 10.1186/s12967-016-0824-x The sequences of siRNA and primers for RT-PCR. [file 12967_2016_824_MOESM2_ESM.docx]

Table S2 The sequences of siRNA and primers for RT-PCR

| **Gene name** | **Sense sequence** | **Anti-sense sequence** | **Product size (bp)** |
| --- | --- | --- | --- |
| HMGA1 | 5'-AGGGAAGATGAGTGAGTCG -3' | 5'-AAGCTGCTCCTCCAGTGAG -3' | 337 |
| E-cadherin | 5’-TCGACACCCGATTCAAAGTGG-3’ | 5’-TTCCAGAAACGGAGGCCTGAT-3’ | 194 |
| Snail | 5'-CGCGAATCGGCGACCCCAGT-3' | 5'-GGTCAGCGAAGGCACGGCTG-3' | 690 |
| β-actin | 5'- ATCTGGCACCACACCT-3' | 5'-CGTCATACTCCTGCTT -3' | 837 |
| HMGA1 siRNA | 5'-GACCCGGAAAACCACCACATT-3' | 5'-UGUGGUGGUUUUCCGGGUCTT-3' |  |
