# Supplementary material for: The association between S100A13 and HMGA1 in the modulation of thyroid cancer proliferation and invasion
Source: J Transl Med. 2016 Mar 23;14:80. doi: 10.1186/s12967-016-0824-x (PMC4804518; doi:10.1186/s12967-016-0824-x)
Supplement: Supplementary file 3 — 10.1186/s12967-016-0824-x Selection of the lentiviral vector harboring shRNA with the highest knockdown efficiency. Relative levels of S100A13 in TPC1 cells infected with different groups of lentiviral particles at a low MOI or a high MOI. Low dose group: The dose of lentiviral plasmid was 0.30 μg; High dose group: The dose of lentiviral plasmid was 0.6 μg. **P < 0.01, vs NC group; n = 3. The highest knockdown efficiency was achieved using KD1-harboring lentiviral particles. [file 12967_2016_824_MOESM3_ESM.pdf]

Figure S1

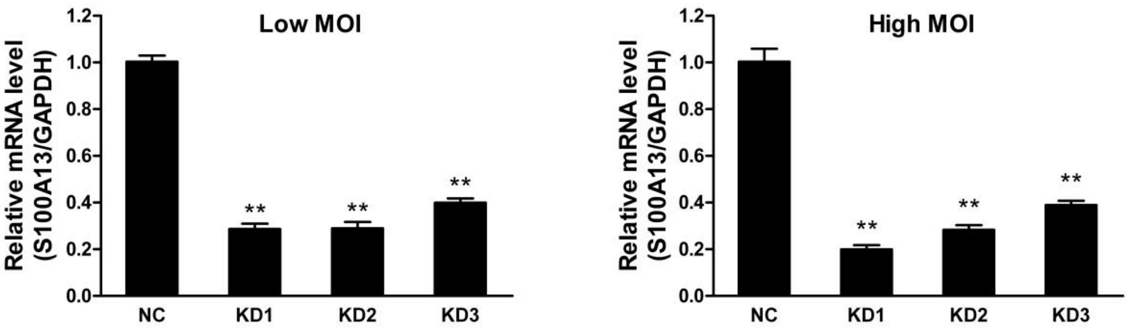

Figure S1 Selection of the lentiviral vector harboring shRNA with the highest knockdown efficiency. Relative levels of S100A13 in TPC1 cells infected with different groups of lentiviral particles at a low MOI or a high MOI. Low dose group: The dose of lentiviral plasmid was 0.30 μg; High dose group: The dose of lentiviral plasmid was 0.60 μg. ***P <0.01*, vs NC group; n =3. The highest knockdown efficiency was achieved using KD1-harboring lentiviral particles.
