# Supplementary material for: The association between S100A13 and HMGA1 in the modulation of thyroid cancer proliferation and invasion
Source: J Transl Med. 2016 Mar 23;14:80. doi: 10.1186/s12967-016-0824-x (PMC4804518; doi:10.1186/s12967-016-0824-x)
Supplement: Supplementary file 5 — 10.1186/s12967-016-0824-x Lentivirus-mediated S100A13 knockdown was utilized to detect the effect on migration capability with scratch-wound assays in TPC1 cell. [file 12967_2016_824_MOESM5_ESM.pdf]

Figure S3

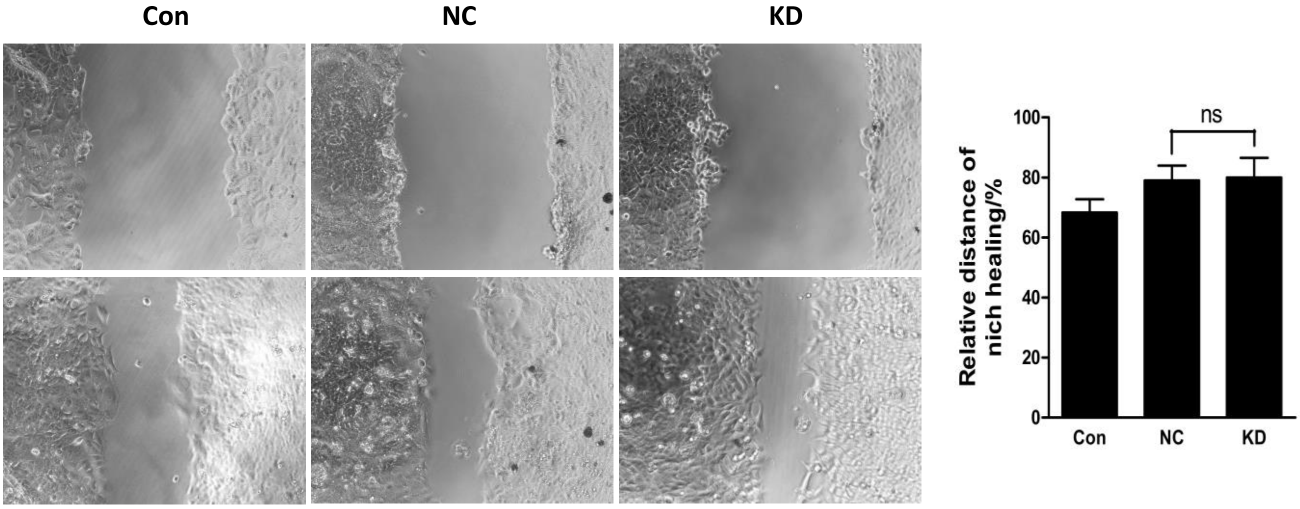

Figure S3 Lentivirus-mediated S100A13 knockdown was utilized to detect the effect on migration capability with scratch-wound assays in TPC1 cell.
