# Supplementary material for: The association between S100A13 and HMGA1 in the modulation of thyroid cancer proliferation and invasion
Source: J Transl Med. 2016 Mar 23;14:80. doi: 10.1186/s12967-016-0824-x (PMC4804518; doi:10.1186/s12967-016-0824-x)
Supplement: Supplementary file 6 — 10.1186/s12967-016-0824-x S100A13 increase the mRNA levels of HMGA1 and SNAIL in TPC-1 cells. The GV219/S100A13 plasmid was introduced into TPC-1 cells for 48 h, and the mRNA levels of HMGA1 and SNAIL were assessed by Q-PCR. Average values of three independent experiments are shown, error bar indicates ±s.d. **p < 0.05. [file 12967_2016_824_MOESM6_ESM.pdf]

Figure S4

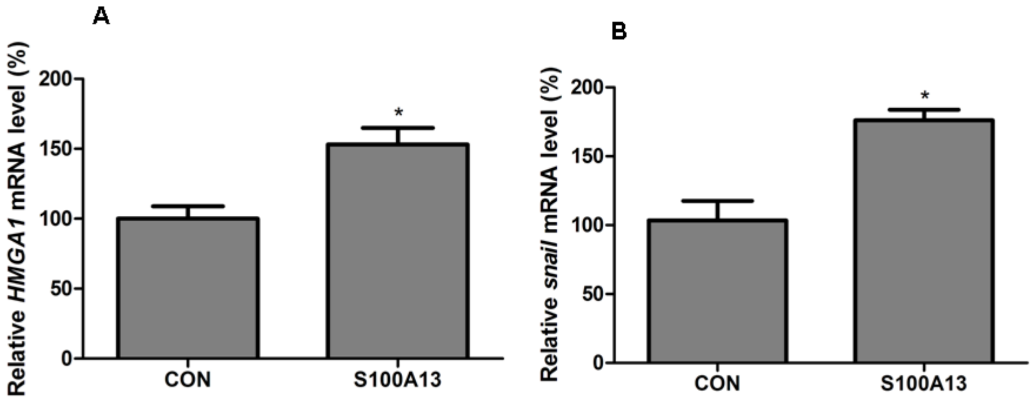

Figure S4 S100A13 increase the mRNA levels of HMGA1 and SNAIL in TPC-1 cells

The GV219/ S100A13 plasmid was introduced into TPC-1 cells for 48h, and the mRNA levels of HMGA1 and SNAIL were assessed by Q-PCR . Average values of three independent experiments are shown, error bar indicates ±s.d. *p<0.05
