# Supplementary material for: The association between S100A13 and HMGA1 in the modulation of thyroid cancer proliferation and invasion
Source: J Transl Med. 2016 Mar 23;14:80. doi: 10.1186/s12967-016-0824-x (PMC4804518; doi:10.1186/s12967-016-0824-x)
Supplement: Supplementary file 7 — 10.1186/s12967-016-0824-x S100A13 increase the promoter activities of HMGA1 and SNAIL in TPC-1 cells. The PGL.10/ HMGA1 and PGL.10/ SNAIL were co-transfected with or without GV219/ S100A13 plasmid into TPC-1 cells. Average values of three independent experiments are shown, error bar indicates ±s.d. *p < 0.05, **p < 0.01. [file 12967_2016_824_MOESM7_ESM.pdf]

Figure S5

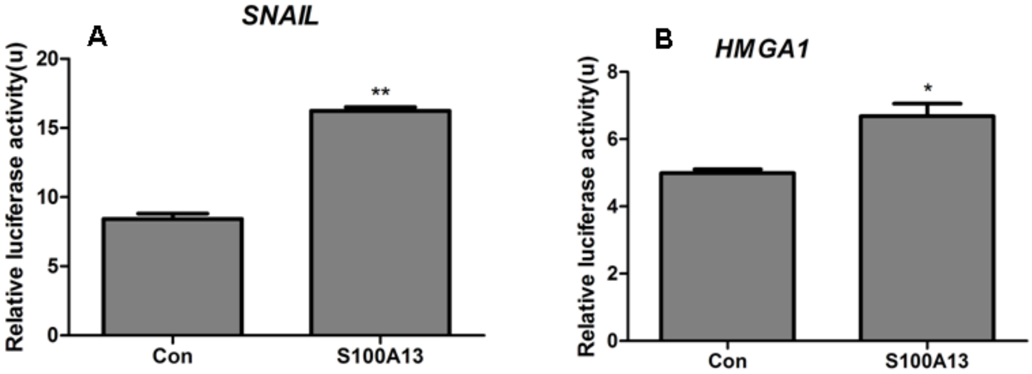

Figure S5 S100A13 increase the promoter activities of *HMGA1* and *SNAIL* in TPC-1 cells

The PGL.10/ HMGA1 and PGL.10/ SNAIL were co-transfected with or without GV219/ S100A13 plasmid into TPC-1 cells. Average values of three independent experiments are shown, error bar indicates ±s.d. *p<0.05, **p<0.01
